# Toward the Assessment of Food Toxicity for Celiac Patients: Characterization of Monoclonal Antibodies to a Main Immunogenic Gluten Peptide

**DOI:** 10.1371/journal.pone.0002294

**Published:** 2008-05-28

**Authors:** Belén Morón, Michael T. Bethune, Isabel Comino, Hamid Manyani, Marina Ferragud, Manuel Carlos López, Ángel Cebolla, Chaitan Khosla, Carolina Sousa

**Affiliations:** 1 Departamento de Microbiología y Parasitología, Facultad de Farmacia, Universidad de Sevilla, Sevilla, Spain; 2 Department of Chemistry, Stanford University, Stanford, California, United States of America; 3 Department of Biochemistry, Stanford University, Stanford, California, United States of America; 4 Biomedal S.L., Sevilla, Spain; 5 Departamento de Biología Molecular, Instituto de Parasitología y Biomedicina “López-Neyra”, Consejo Superior de Investigaciones Científicas (CSIC), Granada, Spain; 6 Department of Chemical Engineering, Stanford University, Stanford, California, United States of America; Centre de Recherche Public-Santé, Luxembourg

## Abstract

**Background and Aims:**

Celiac disease is a permanent intolerance to gluten prolamins from wheat, barley, rye and, in some patients, oats. Partially digested gluten peptides produced in the digestive tract cause inflammation of the small intestine. High throughput, immune-based assays using monoclonal antibodies specific for these immunotoxic peptides would facilitate their detection in food and enable monitoring of their enzymatic detoxification. Two monoclonal antibodies, G12 and A1, were developed against a highly immunotoxic 33-mer peptide. The potential of each antibody for quantifying food toxicity for celiac patients was studied.

**Methods:**

Epitope preferences of G12 and A1 antibodies were determined by ELISA with gluten-derived peptide variants of recombinant, synthetic or enzymatic origin.

**Results:**

The recognition sequences of G12 and A1 antibodies were hexameric and heptameric epitopes, respectively. Although G12 affinity for the 33-mer was superior to A1, the sensitivity for gluten detection was higher for A1. This observation correlated to the higher number of A1 epitopes found in prolamins than G12 epitopes. Activation of T cell from gluten digested by glutenases decreased equivalently to the detection of intact peptides by A1 antibody. Peptide recognition of A1 included gliadin peptides involved in the both the adaptive and innate immunological response in celiac disease.

**Conclusions:**

The sensitivity and epitope preferences of the A1 antibody resulted to be useful to detect gluten relevant peptides to infer the potential toxicity of food for celiac patients as well as to monitor peptide modifications by transglutaminase 2 or glutenases.

## Introduction

Celiac disease (CD) is a common autoimmune disorder that has genetic, environmental, and immunological components. Though under-diagnosed, it is one of the most prevalent chronic gastrointestinal diseases in humans, and exhibits unusually large clinical, histological, immunological, and genetic heterogeneity [1], [2]. The clinical spectrum of CD has been expanded in recent years, with the identification of asymptomatic patients, patients with minimal symptoms (the most difficult to detect), and patients with extra-intestinal symptoms [2]–[5]. Regardless of symptomatic presentation, active disease in virtually all CD patients relies on dietary exposure to a common environmental antigen, gluten. The ingestion of gluten proteins contained in wheat, barley, and rye, and, in some cases, oats [6], [7], leads to characteristic inflammation, villous atrophy, and crypt hyperplasia in the CD patient upper small intestine [2].

In wheat gluten, the principal toxic components belong to a family of closely related proline and glutamine rich proteins called gliadins [8]. Several epitopes responsible for the toxicity of gliadins have been identified based on their ability to stimulate proliferation of gluten-responsive DQ2 (or DQ8) restricted CD4+ T cells in CD patient-derived small intestine biopsies [9]–[12]. To elicit a T-cell response, most gliadin epitopes must undergo a transglutaminase 2-mediated deamidation of certain glutamine residues to glutamate residues [10].

Among the main dietary proteins, gluten is unique in that it contains approximately 15% proline and 35% glutamine residues [13]. This high proline and glutamine content prevents complete proteolysis by gastric and pancreatic enzymes, such that long oligopeptides that are toxic to CD patients build up in the small intestine. One peptide in particular, the 33-mer from α-2 gliadin (residues 57–89), contains 6 T-cell epitopes, is highly proteolytically resistant, and is a principal contributor to gluten immunotoxicity [14]. It has been proposed that oral administration of a therapeutic dose of a suitably formulated prolyl endopeptidase (SC PEP from *Sphingomonas capsulata*) and glutamine specific endoprotease (EP-B2, a cysteine endoprotease from germinating barley seeds) might counter the toxic effects of certain quantities of ingested gluten [15]–[20].

At present, the prescription of a gluten-free diet is the only therapy for CD patients. However, it is not easy to maintain a diet with zero gluten content because gluten contamination in food is commonplace [21]. Gluten is a common ingredient in the human diet; after sugar, it is perhaps the second most widespread food substance in Western civilization. Since about 10% of gluten seems to be made up of potentially toxic gliadin peptides [22], it is desirable to quantify the amount of these peptides ingested by a CD patient, so that the factual toxicity of the gluten present in foods can be established more precisely. In a previous work, we obtained monoclonal antibodies (moAb) (G12 and A1) against the gliadin 33-mer peptide [23]. The aims of the current study were two-fold. First, we sought to characterize the sequence specificity of these anti-33-mer moAbs using a panel of overlapping fusion peptides based on the 33-mer sequence. Second, we evaluated the practical efficacy of these moAbs as analytical tools for quantifying food toxicity for CD patients. Toward this end, we showed that the reactivity of each moAb with a variety of cereal storage proteins correlated with the immunotoxicity of those dietary grains from which the proteins were extracted. Additionally, we showed with one of these moAb (A1) that reductions in the 33-mer content of commercial whole-wheat bread caused by treatment with candidate therapeutic glutenases can be quantified using a simple, high-throughput competitive ELISA. Our results establish A1 and G12 anti-33-mer moAbs as specific and reliable tools for detecting foods potentially harmful for CD patients.

## Results

### Detection of gliadin immunogenic peptide by anti-33-mer moAbs

**Figure 1:**
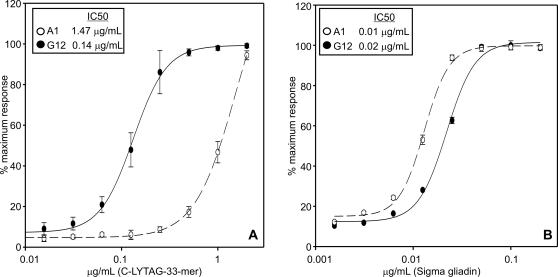
Standard curve of the detection of C-LYTAG-33-mer polypeptide (A) and Sigma gliadin (B) by indirect ELISA with use of moAbs G12 (black) and A1 (white). Each point of the curve represents the mean±standard deviation of n = 4 assays. IC50 values of the moAbs to both antigens are indicated.

The 33-mer peptide from α-2 gliadin is a principal contributor to gluten immunotoxicity [14]. Thus the production of moAbs against this toxic gluten peptide could be of great importance in both research and diagnosis. In a previous work, we obtained moAbs against the 33-mer peptide (A1 and G12 moAbs) [23]. To test the relative sensitivity of each moAb for the 33-mer peptide, we immobilized different concentrations of the C-LYTAG-33-mer polypeptide, and detected with A1 and G12 moAb in an indirect ELISA. The affinity of each moAb for the antigen was quantified by calculation of the concentration of the antigen giving a 50% reduction of the peak signal in the ELISA (IC50). The sensitivity of the G12 moAb for the toxic 33-mer peptide was about eight times higher than that of A1 (Figure 1A). To test for moAb specificity, we studied the cross-reactivity values (CR) of these moAb against commercial gliadin, also by indirect ELISA. The G12 moAb presented an IC50 of almost double that obtained with the A1 moAb, suggesting that A1 had broader reactivity with gliadin epitopes than G12, which is more specific for the 33-mer (Figure 1B).

### Characterization of A1 and G12 moAbs sensitivity for celiac-toxic cereals

In a previous work, we investigated whether the G12 moAb was able to detect the presence of gliadin 33-mer related epitopes in prolamins from various cereals [23]. The results indicated that the moAb showed CR against prolamins of wheat, barley, rye and oats that are toxic for CD patients [23]. Since the A1 moAb showed even higher sensitivity to wheat gliadin than G12, we evaluated the reactivity of A1 moAb with other cereal grain prolamins. The prolamins from wheat, barley, rye, oats, corn, and rice were extracted, and the samples were analyzed by Western blot, using the A1 moAb. The A1 moAb detected wheat gliadins, barley hordeins, and rye secalins. Oat avenins were also detected by A1, but the sensitivity obtained was lower (data not shown). The A1 moAb did not react to a detectable extent with prolamins extracted from rice (oryzein) and corn (zein), cereals that are non-toxic to CD patients.

**Figure 2:**
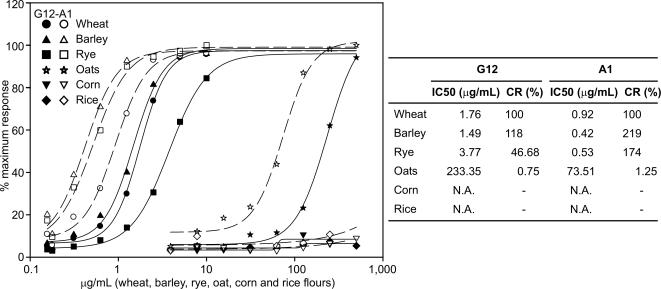
Comparative reactivity of prolamins from wheat, barley, rye, oats, corn and rice from indirect ELISAs using moAbs G12 (black) and A1 (white). Each point of the curve shows the mean of n = 3 assays. IC50 and CR values of the moAbs to prolamins are indicated. N.A.: Not applicable.

To obtain quantitative data about the capacity of A1 to detect celiac toxic prolamins, we performed an indirect ELISA with samples of wheat, barley, rye, oats, rice, and corn (Figure 2). The assay proved to be highly specific for wheat, rye and barley, as no signal was observed in samples containing prolamins from rice or corn (Figure 2). The G12 and A1 moAbs detected oats with lower sensitivity, indicating that there are peptides in avenin with sequence similarity to the 33-mer. This is consistent with the identification of proline and glutamine rich epitopes in avenins that are toxic in some CD patients [6]. The lower sensitivity for oat avenins may be due to the lower proportion of oat flour protein content that comprises prolamins relative to the proportion of gliadins, hordeins or secalins in their respective grains [24].

The A1 moAb was clearly more sensitive than the G12 moAb for the detection of the prolamin fractions from wheat, barley, rye, and oats. Although they were targeted at the toxic 33-mer peptide of wheat gliadin, both moAb were more sensitive for barley than for wheat, and the A1 was also more sensitive for the prolamins of rye than for those of wheat. Although both moAb were more sensitive for barley than for wheat (Figure 2), the A1 moAb had almost three-fold higher affinity for barley than did G12.

### Development of a competitive ELISA assay using the A1 moAb

The preparation of many foodstuffs involves heating or enzymatic processes that may partially hydrolyze or deamidate gluten. As a result, the quantity of gluten extracted from foodstuffs processed by heat or fermentation may be underestimated by indirect or sandwich ELISA [25], [26]. We therefore developed a competitive ELISA in which the antigen was fixed on the plate and soluble antigens in the sample, were preincubated with a HRP conjugated moAb. Upon addition of this preincubated mixture to the plate, soluble and fixed antigens competed for binding to the HRP conjugated moAb. Thus, a decrease in the signal indicates the presence of antigen in the sample. A competitive ELISA method using G12 moAb was previously developed for detecting toxic peptides in hydrolyzed food down to 0.5 ppm gliadin [23]. Here we developed a similar assay using the A1 moAb.

Several assays were carried out to optimize the conditions for the competitive ELISA, including altering the concentration of gliadin fixed on the plate (1–0.01 µg/well), the dilution of A1-HRP moAb used (1∶1,000–1∶50,000), and the time (15 min to 4 h) and temperature (4°C, room temperature, and 37°C) of preincubation and incubation. A concentration of 0.5 µg/well of gliadin fixed on the plate and a 1∶10,000 dilution of the A1-HRP moAb were found to be optimal (data not shown). A preincubation of 3 h and an incubation of 40 min, both at room temperature, were found to be optimal. The resulting highly sensitive competitive assay had a limit of detection of 1.63 ng/mL of gliadin (0.33 ppm of gluten) and a limit of quantification of 6.98 ng/mL of gliadin. IC50 in the standard curve of the competitive assay was 15.76 ng/mL. The standard curve for the detection of toxic gliadin by competitive ELISA under the established conditions showed a good correlation to the data (R^2^ = 0.99).

The repeatability and reproducibility of the method, calculated from various standard curves performed on the same ELISA plate (intra-assay), and on different ELISA plates (inter-assay), were determined. The intra-assay coefficient of variation of the standards situated between 100 and 1.56 ng/mL of gliadin was found to be between 1.37% and 5.21%, while the inter-assay coefficient of variation was between 3.16% and 11.78% for the same standards.

### Analysis of the epitope recognition of G12 and A1 moAb

**Figure 3:**
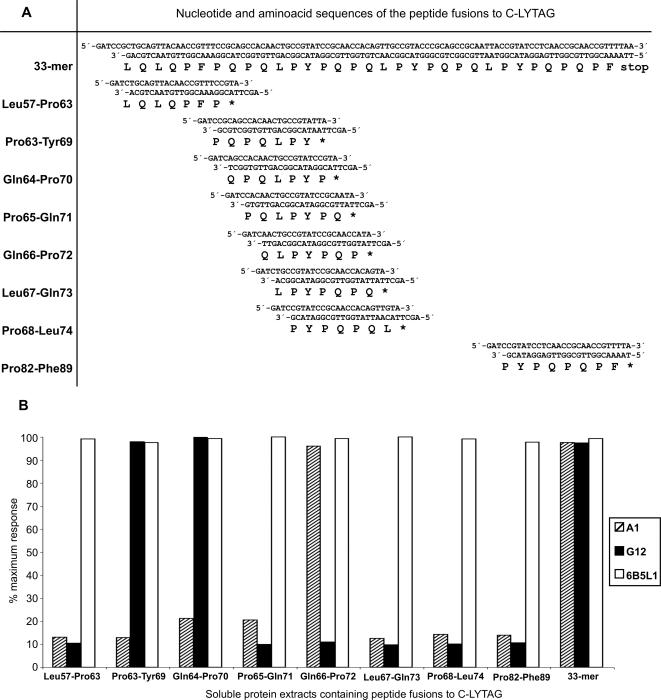
Analysis of anti-33-mer moAbs recognition regions in recombinant 33-mer peptide fragments expressed in *E. coli*. A. Nucleotide sequences and the deduced amino acid sequences of the encoded peptide fusions to C-LYTAG. B. Detection of C-LYTAG-peptide fusions by an indirect ELISA with the use of moAbs G12, A1 and 6B5L1.

To determine the epitope recognized by the G12 and A1 moAb within the 33-mer peptide, fusions of the C-LYTAG coding sequence of the pALEXb plasmid (Biomedal S.L., Sevilla, Spain) were constructed with coding sequences of hepta- and octapeptides comprising the complete sequence of the 33-mer peptide (Figure 3A). The resulting plasmids were introduced by transformation into the REG1 strain of *Escherichia coli*, allowing for over-expression of the encoded fusion proteins upon induction (Biomedal S.L., Sevilla, Spain). The over-expressed bacterial extracts were analyzed by indirect ELISA using the anti-33-mer A1 and G12 moAbs. Similarly, the anti-C-LYTAG 6B5L1 moAb (Biomedal, S.L., Sevilla, Spain) was used to establish that the designed protein was expressed intact in all cases. A reference signal in the bacterial extract containing the C-LYTAG-33-mer fusion protein was observed for all the moAbs assayed (A1, G12 and 6B5L1) (Figure 3B). Saturating signals were obtained in the indirect ELISA analysis using the anti-C-LYTAG 6B5L1 moAb for all the analyzed fusion proteins, indicating that all fusion proteins were over-expressed (Figure 3B).

With regard to the determination of the sequence of recognition of the anti-33-mer moAbs (G12 and A1), a positive signal was detected only in the bacterial extracts containing the fusion peptides Pro63-Tyr69 (PQPQLPY) and Gln64-Pro70 (QPQLPYP) for the G12 moAb and in the bacterial extracts containing the fusion peptide Gln66-Pro72 (QLPYPQP) for the A1 moAb (Figure 3B). These results thus indicate that the region of recognition within the 33-mer peptide for the G12 moAb is QPQLPY (common to the fusion proteins Pro63-Tyr69 and Gln64-Pro70) and that for the A1 moAb is QLPYPQP.

### Study of the relative affinity of the G12 moAb for different peptide variants derived from the regions of recognition

**Figure 4:**
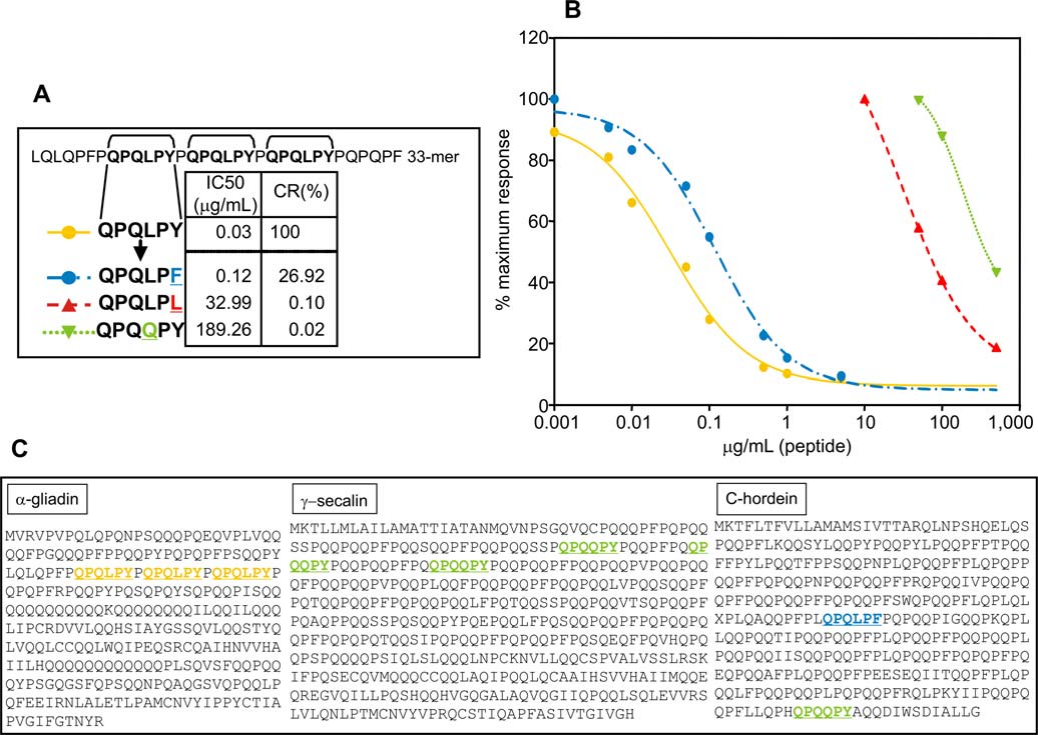
Relative affinity of moAb G12 for different peptide variants derived from its recognition region (QPQLPY). A. Amino acid sequences of the peptides. The G12 recognition sequence in the 33-mer peptide is in bold face. IC50 and CR values of the moAb G12 to peptides are indicated. B. Competition assay measuring the affinity of the moAb G12 for the peptides. Two separate assays were performed with the antibody, each with three repetitions. C. Localization of the peptides in the α-gliadin (accession number: JQ1047), γ-secalin (accession number: ABO32294.1) and C-hordein (accession number: AAA92333.1) sequences. The same color code for labelling the peptides has been used in A, B and C.

The recognition sequence of the G12 moAb (QPQLPY) is repeated three times within the gliadin 33-mer peptide. To determine the relative affinity of G12 for this epitope, and for similar sequences present elsewhere in toxic prolamins, we constructed hexapeptide variants of the G12 epitope, two of which were designed based on their presence in the prolamins of barley and rye (Figure 4A and 4C). The affinity of the G12 moAb for different hexapeptide variants was determined in a competitive assay in which immobilized gliadin was challenged with QPQLPY-derivative peptides as soluble competitors (Figure 4A). The G12 moAb had high affinity for the peptide QPQLPF, reduced only four-fold relative to the previously identified epitope recognized by this moAb in the 33-mer, QPQLPY (Figure 4B). While the conservative replacement of tyrosine (QPQLPY) with phenylalanine (QPQLPF) did not drastically reduce the affinity of the G12 moAb, substitution with leucine (QPQLPL) reduced the affinity a thousand-fold, indicating the importance of this last position in determining affinity. A dramatic reduction in affinity was also observed for the peptide QPQQPY, such that the affinity of the anti-33-mer G12 moAb decreased as follows: QPQLPY>QPQLPF≫QPQLPL>QPQQPY.

### Determination of the peptide sequence preferences for moAb A1 binding

**Figure 5:**
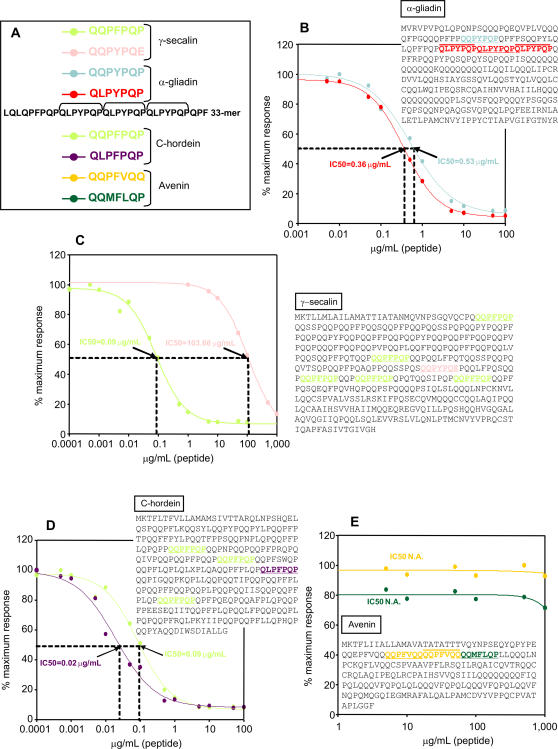
Relative affinity of moAb A1 for different peptide variants derived from its recognition region (QLPYPQP). A. Amino acid sequences of the peptides. The A1 recognition sequence in the 33-mer peptide is in red. B, C, D and E. Competition assay for detection of the affinity of the moAb A1 for the peptides and their localization in α-gliadin (B; accession number: JQ1047), γ-secalin (C; accession number: ABO32294.1), C-hordein (D; accession number: AAA92333.1) and avenin (E; accession number: AAA32716.1). Two separate assays were performed with the moAb, each with three repetitions. IC50 values of the moAb A1 to peptides are indicated. N.A.: Not applicable. The color code for labelling the peptides is the same as that used in A.

We also studied the relative affinity of the A1 moAb for its recognition sequence (QLPYPQP) and for related peptide variants by a competitive assay (Figure 5A). The peptides assayed for A1 were more numerous than for G12 due to the longer heptapeptide recognition sequence recognition contained in the 33-mer and due to the suspected broader specificity of A1 for other prolamin sequences based on indirect ELISA assays (Figure 2). Figure 5 shows the affinity of the A1 moAb for the different peptides assayed; the IC50 was used to compare the affinity of A1 for each peptide. Notably, two peptides present in secalin and hordein (QQPFPQP and QLPFPQP, Figures 5C and 5D, respectively) showed higher affinity for the A1 moAb than did the 33-mer-derived recognition sequence peptide (QLPYPQP). This suggests that the fourth residue in the recognition sequence is substantially important to A1 recognition, whereas the second position is not. Consistent with this, gliadin peptides QLPYPQP and QQPYPQP showed comparable affinity for the moAb (Figure 5B). The affinity of the anti-33-mer A1 moAb for epitopes present in celiac-toxic cereals decreased as follows: QLPFPQP>QQPFPQP>QLPYPQP>QQPYPQP≫QQPYPQE.

The affinity for the sequence included in the wheat gliadin 33-mer was not as high as for QQPFPQP, which is one of the most abundant sequences in secalin and hordein, similar to the 33-mer epitope in gliadin. This may explain why, despite its lower affinity for 33-mer peptide relative to the G12 moAb, the A1 moAb had higher sensitivity for the whole range of toxic cereals tested in this study. The A1 moAb may therefore be useful as a sensitive detection tool for identifying celiac-toxic peptides in complex foodstuffs.

Preliminary attempts to find an avenin epitope gave no positive results (Figure 5E). The prolamins in oats represent much less of the total seed proteins than in the other cereals [24]. Furthermore, the amount of proline residues contained in avenins (10%) is about two-thirds that in the prolamins of wheat (gliadins and glutenins), barley (hordeins), and rye (secalins). In any case, we tested certain previously proposed potential avenin epitopes located in the avenin regions with the highest content of proline residues, regions also rich in glutamine, but could not obtain any reactivity to the A1 moAb.

**Figure 6:**
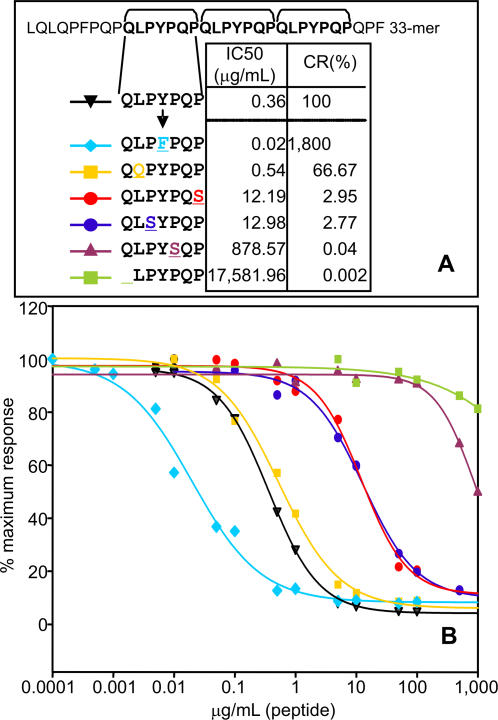
Relative affinity of moAb A1 for peptide variants of the 33-mer recognition region (QLPYPQP) featuring single amino acid sustitutions. A. Amino acid sequences of the peptides. The A1 recognition sequence in the 33-mer peptide is in bold face. IC50 and CR values of the moAb A1 to peptides are indicated. B. Competition assay for the detection of the affinity of the moAb A1 for the peptides. Two separate assays were performed with the antibody, each with three repetitions. The color code for labelling the peptides is the same as that used in A.

To study the relative importance of glutamine and proline residues in epitope selection by the A1 moAb, single substitutions or deletions were made to these amino acids in the recognition sequence (QLPYPQP; Figure 6A). We performed the analysis with the A1 moAb rather than with the G12 moAb because A1 has higher sensitivity for prolamins from toxic cereals. When the first glutamine of the A1 recognition sequence was eliminated (LPYPQP), the affinity for A1 decreased significantly, consistent with the results from epitope scanning with the C-LYTAG fusions (Figure 3). Substitutions of each proline residue in the recognition sequence with a serine residue decreased A1 affinity markedly (Figures 6A and 6B). This effect was most marked when the substitution was made in the second proline position (QLPY**S**QP), resulting in a CR that was practically zero.

These results indicate that the initial glutamine residue and all three prolines of the epitope QLPYPQP were important for its recognition by A1, suggesting that this moAb could serve as a tool for monitoring enzymatic degradation of toxic peptides by potentially therapeutic glutamine and proline specific proteases.

### Use of moAb A1 to monitor gluten detoxification by candidate glutenases

Oral administration of glutamine and proline specific proteases (i.e. glutenases) represents a potential therapeutic alternative (or adjunct) to a gluten-free diet [27], [28]. However, validation of the efficacy of these enzymes at detoxifying gluten *in vitro* must precede clinical testing, and such validation currently relies on low-throughput, technically challenging cell culture-based assays [19], [20], [27] or on polyclonal anti-gliadin antibody-based ELISA assays that are only grossly quantitative [20]. A competitive ELISA using a anti-33-mer moAb would enable high-throughput, highly quantitative testing of gluten detoxification by candidate therapeutic glutenases.

**Figure 7:**
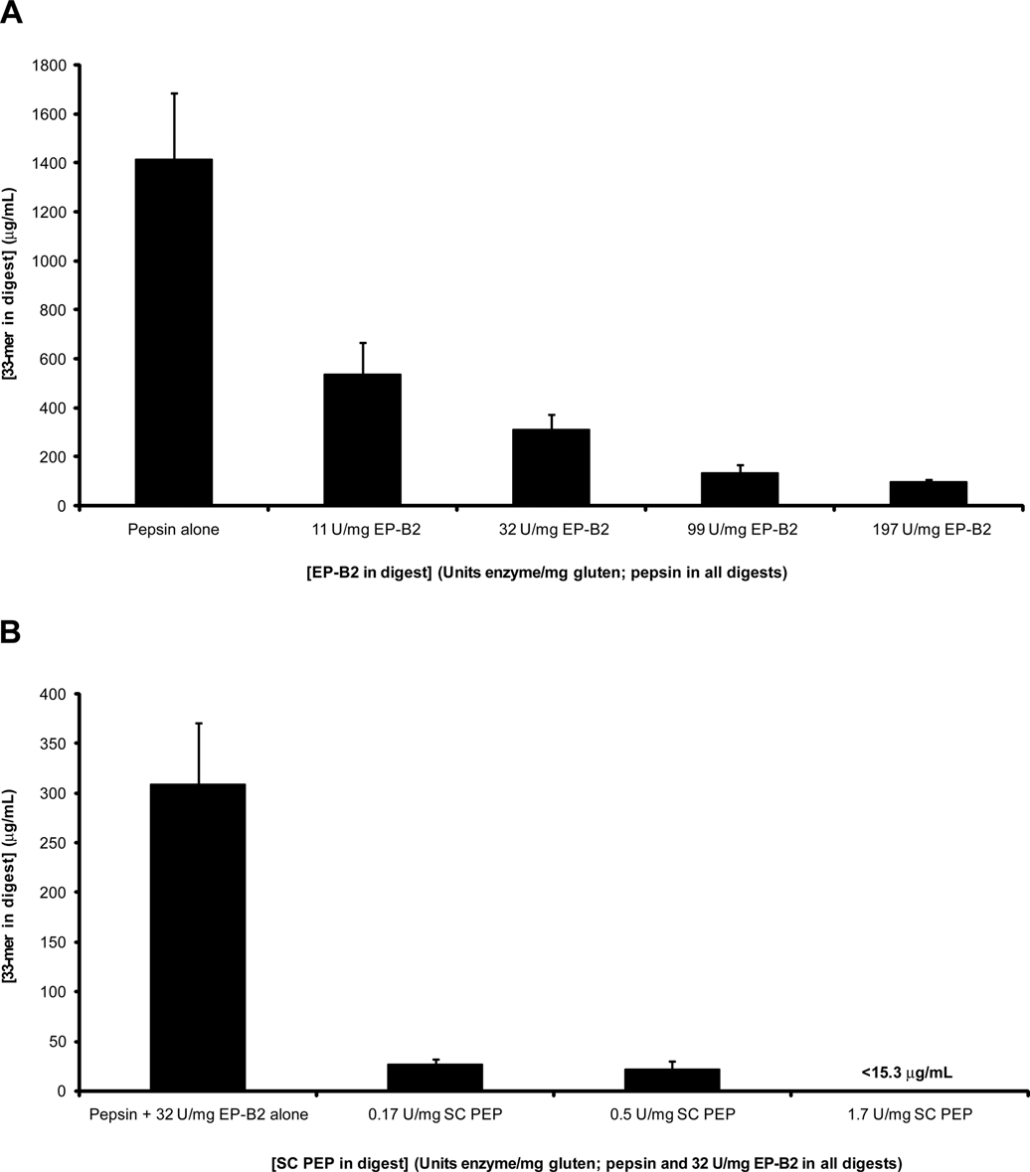
Indirect competitive ELISA using moAb A1 to test whole-wheat bread digests for 33-mer content. A. Concentration of 33-mer (µg/mL) in whole-wheat bread digests containing 0.6 mg/mL pepsin supplemented with specified concentrations of recombinant proEP-B2 (U/mg gluten). B. Concentration of 33-mer (µg/mL) in whole-wheat bread digests containing 0.6 mg/mL pepsin and 32 U/mg EP-B2 supplemented with specified concentrations of recombinant SC PEP (U/mg gluten). The concentration of 33-mer in each digest was determined by comparison to a synthetic 33-mer standard curve. Two separate assays were performed with the antibody, each with three repetitions.

We digested commercial whole-wheat bread under mock gastric conditions for 60 min with pepsin supplemented either with EP-B2 at varied concentrations (Figure 7A), or with a fixed EP-B2 concentration plus varied concentrations of SC PEP (Figure 7B). Dilution series of the quenched digests were prepared in parallel with a calibration dilution series of chemically synthesized 33-mer peptide, and these were tested against fixed 33-mer in an indirect competitive ELISA using moAb A1. Treatment of whole-wheat bread with EP-B2 reduces the concentration of the 33-mer and close analogs by up to 10-fold in a dose-dependent manner (Figure 7A). This is consistent with the observation that EP-B2 cleaves the 33-mer after Gln66, Gln73, and Gln80 [17], cleavages expected to extirpate the affinity of A1 for the resultant fragments (Figures 3 and 6). The combination of EP-B2+SC PEP further reduced antigen concentrations by at least an additional 10-fold to levels undetectable by our methods (Figure 7B). This is again consistent with previously published results, in which EP-B2 substantially detoxified similar bread digests, but the synergistic combination of EP-B2 with SC PEP was required to dramatically reduce the intestinal T cell reactivity of these digests [20]. The intensity of the signal obtained with the A1 moAb in our assay was therefore proportional to the potential damage caused to a CD patient by a commercial gluten source.

### Analysis of the recognition of anti-33-mer moAbs for deamidated and innate gluten peptides

**Figure 8:**
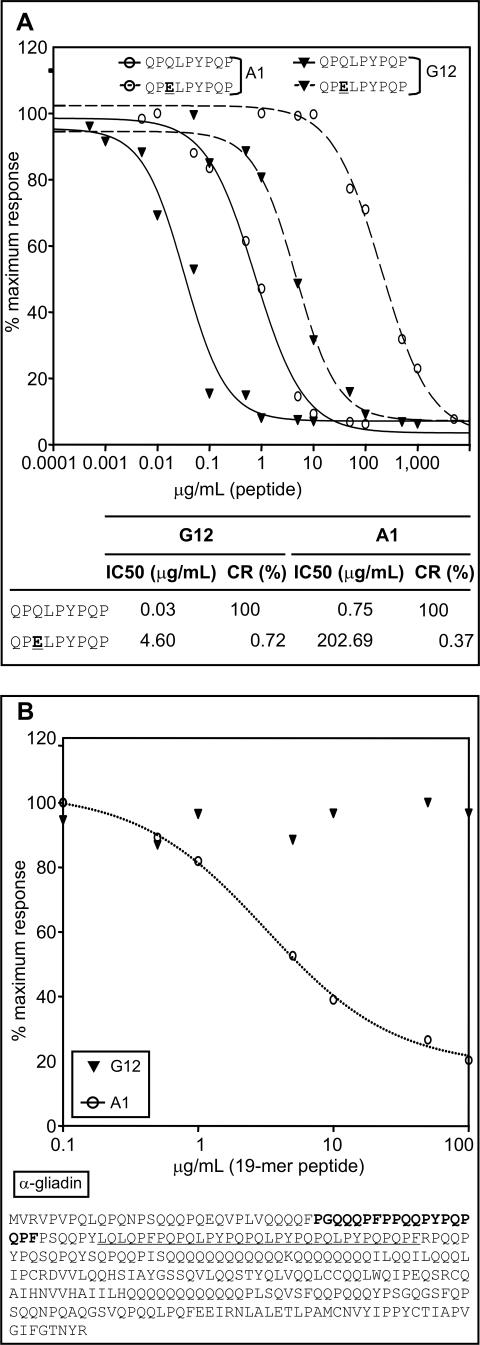
Relative affinity of the anti-33-mer moAbs for different peptides involved in the immune responses to gluten in CD patients. A. Competition assay for detection of the affinity of G12 and A1 moAbs for a peptide containing recognition common regions (QPQLPYPQP) to the two moAbs and its deamidated counterpart (QPELPYPQP). IC50 and CR values of the anti-33-mer moAbs to peptides are indicated. B. Competition assay for detection of the affinity of the moAbs G12 and A1 for the innate peptide, p31-49. The 33-mer and the p31-49 peptide sequences are underlined and in bold face, respectively. Wheat alpha-gliadin (α-gliadin, accession number: JQ1047). Two separate assays were performed with the antibodies, each with three repetitions (A and B).

CD is closely associated with genes that code for human leukocyte antigens DQ2 and DQ8. These have been shown to bind with high affinity to gliadin-derived peptides in which specific glutamine residues in key positions have been converted to glutamic acid by transglutaminase 2-mediated deamidation [1], [16]. A moAb capable of discriminating between native and deamidated gluten peptides would be a valuable research tool for monitoring the fate of digested prolamin peptides. To test the relative sensitivity of each moAb for the deamidated 33-mer peptide, a peptide (QPQLPYPQP) was designed that represented a region of recognition common to the two moAbs, together with the same peptide deamidated (QPELPYPQP) (Figure 8A). The affinities of the A1 and G12 moAbs for these peptides were determined by a competitive assay in which immobilized gliadin was challenged with peptides as competitors. The affinity of the G12 moAb for the deamidated peptide was about forty times higher than that of the A1 moAb (Figure 8A). However, both moAbs recognized the non-deamidated peptide with >100-fold greater affinity than they did the deamidated peptide (Figure 8A). In combination with a previously characterized, commercially available moAb that has 20-fold greater affinity for the deamidated form of an overlapping gluten peptide than for its non-deamidated counterpart [29], moAbs G12 and A1 may be useful for future studies on transglutaminase 2-mediated gluten peptide deamidation.

The innate immune response to gluten plays a key role in the development of CD [30], [31]. This response is mediated by interleukin 15 (a typical cytokine of the innate immune system) and elicited by the toxic peptide, p31-49 (19-mer), derived from alpha-gliadin [31]. To test whether the anti-33-mer A1 and G12 moAbs recognized peptide p31-49, competitive ELISAs with each moAb were performed. The A1 moAb was able to detect p31-49 (IC50 3.18 µg/mL) (Figure 8B). The G12 moAb showed no affinity for the 19-mer peptide (Figure 8B). These results were consistent with our previous identification of the QQPYPQP peptide, included in p31-49, as a permissive epitope for the A1 moAb (Figure 5B). Therefore, this moAb shows an interesting range of peptide recognition that includes gliadin peptides involved in the both the adaptive and innate immunological responses in CD.

## Discussion

In this work, we characterized two moAbs raised against the immunotoxic gliadin 33-mer peptide, each of which showed suitable epitope recognition for fundamental and practical applications in CD research. The most straightforward of these is that these moAbs may be used to detect immunotoxic gluten epitopes in foodstuffs via immunological assays. In a previous work we obtained moAbs (G12 and A1) against the 33-mer, a peptide from α-2 gliadin that is a principal contributor to gluten immunotoxicity [14]. Here, we characterized the precise site of peptide recognition and the practical efficacy of these moAbs as analytical tools for quantifying food toxicity for CD patients. ELISAs based on moAbs G12 and A1 exhibited broad specificity toward prolamins toxic to CD patients, along with a high degree of sensitivity, accuracy, and reproducibility. The A1 moAb was particularly sensitive to immunotoxic prolamins, with highest affinity for barley and rye, followed by wheat. The G12 moAb was more specific for the 33-mer than was A1, but it also recognized all immunotoxic prolamins, with highest affinity for barley and wheat, followed by rye. Both moAbs detected oat prolamins, albeit with substantially reduced sensitivity. To provide a basis for these observed sensitivities, we identified the hexapeptide (QPQLPY) and heptapeptide (QLPYPQP) epitopes recognized by G12 and A1 moAbs, respectively, and studied the relative affinity of these moAbs for peptide variants of their recognition sequence. This analysis suggested that A1 showed higher affinity for prolamins of wheat, barley, rye and oats than did G12 as a consequence of the prevalence of the A1 recognition sequence and related sequences in these cereals.

The ability of G12 and A1 to detect oats is of potential practical importance. Oats cause damage to the mucosa in a subset of CD patients [2], [6]. In contrast to previously described anti-prolamin moAbs (e.g. R5 and anti-glia-α2/9) [32], [33], G12 and A1 both detected oats although with lower sensitivity than the prolamins from wheat, barley and rye. Peptides recognized by these moAbs were confirmed by western blot (data not shown), exhibiting a pattern not indicative of contamination by other cereals. The reduction in G12 and A1 sensitivity toward oat prolamins relative to immunotoxic prolamins from wheat, rye, and barley may be due to the lower prolamin content in total oat seed protein compared to the other cereals [24]. Moreover, the potential sequences for moAb recognition could be less prevalent in avenins than in gliadins, hordeins, and secalins. Regardless, ELISA assays using G12 and A1 exhibited high sensitivity toward toxic wheat, rye, and barley prolamins; reduced but significant sensitivity toward oat prolamins, which are moderately toxic in a subset of CD patients; and no sensitivity toward non-toxic prolamins from corn and rice. Together, these observations reveal a direct correlation between assay signal and potential toxicity of food samples for CD patients.

This correlation suggested that an ELISA using a moAb specific for immunotoxic gluten epitopes could be used to quantitatively monitor the enzymatic detoxification of gluten. The use of glutamine and proline specific proteases (EP-B2 and SC PEP, respectively) is currently being explored as an oral therapy for CD [15]–[20]. Because our results suggested that the initial glutamine residue and all three prolines in the recognition sequence for A1 were substantially important for A1 recognition, we used this moAb to determine the concentration of immunotoxic epitopes remaining in wheat bread after its treatment with varying amounts of EP-B2 and SC PEP. Decreases in A1 epitopes due to enzymatic digestion by glutenases were consistent with previously reported decreases in T cell activation caused by identical enzymatic treatment of wheat bread [20]. Therefore, the A1 moAb immunoassay may be used to monitor gluten detoxification in enzymatic therapy studies by detecting that part of the digested food that remains toxic for a CD patient.

The 33-mer gliadin peptide and other main prolamin epitopes are deamidated at specific glutamine residues by transglutaminase 2, considerably enhancing their affinity for HLA DQ2 or DQ8 molecules [10], [12]. This transformation is suggested to be critical toward rendering gluten immunotoxic. We determined the affinities of G12 and A1 moAbs for a synthetically deamidated gluten peptide, and found that both moAbs recognized the deamidated peptide with >100-fold less affinity than they did the non-deamidated peptide. These moAbs may be useful for fundamental studies on the mechanism of transglutaminase 2 mediated deamidation either *in vitro* or *in vivo* by discriminating between native and deamidated gluten peptides. Additionally, these moAbs may be used to test the extent to which microbial transglutaminase treatment can abolish gluten toxicity by blocking immunotoxic peptide deamidation sites [34].

Finally, we reported the development of a novel competitive assay using A1, a moAb raised against a 33-mer peptide recognized *in vivo* to be immunotoxic toward patients with CD. A competitive assay is well-suited for food analysis and monitoring of gluten digestion because it can detect both intact proteins and small protein fragments, the latter of which can be underestimated by a sandwich ELISA [32], [35]. The ELISA systems described here showed high reproducibility and repeatability with coefficients of variation below 15%. Taking the minimum working dilution as 1∶10, and starting from a sample (obtained by ethanolic (60%) extraction from a foodstuff) at a concentration of 0.1 g/mL of extraction solution, the A1 competitive ELISA developed would enable the detection of the presence of gluten in foodstuffs at values as low as 0.33 ppm of gluten, which is far below the 20 ppm threshold proposed by the Codex Alimentarius Commission [36]. The recommended maximum daily ingestion of gluten is below 50 mg gluten per day. The method reported here could detect several orders of magnitude less concentration than the maximum recommended gluten concentration in the digestive tract (<20 mg/L) for CD patients [37]. The specificity for immunotoxic peptides obtained by developing moAbs G12 and A1 against the immunotoxic 33-mer is an additional advantage of this immunoassay, since anti-gliadin moAbs with no specificity for toxic peptides may produce false negative or positive signals not related to the remaining toxicity of hydrolyzed food. This work suggests that competitive immunoassays using immunotoxic gluten epitope-specific moAbs, such as G12 and A1, are particularly effective at evaluating food safety for CD patients, with likely advantages over assays measuring the amount of any gluten in a given sample, without regard to its immunotoxicity.

## Materials and Methods

### Materials

Commercially available flours from cultivars of wheat, barley, rye, oats, corn, and rice were employed in this study.

The peptides QPQLPYPQP, QPELPYPQP, QPQLPY, QPQLPF, QPQLPL, QPQQPY, QLPYPQP, QQPYPQP, QQPFPQP, QQPYPQE, QLPFPQP, QQPFVQQ, QQMFLQP, QLPYPQS, QLSYPQP, QLPYSQP and LPYPQP were supplied by Biomedal S.L. (Sevilla, Spain). The 19-mer peptide PGQQQPFPPQQPYPQPQPF was provided by Eduardo Arranz (Universidad de Valladolid-C.S.I.C., Valladolid, Spain).

Whole-wheat bread (Alvarado St Sprouted Whole-Wheat Bread) was from Alvarado St Bakery (Rohnert Park, CA). The synthetic 33-mer peptide used to calibrate the competitive ELISA on bread digests was synthesized using Boc/HBTU chemistry on solid-phase as previously described [38]. Pepsin was purchased from American Laboratories (Omaha, NE). EP-B2 and SC PEP were prepared as previously described [15], [17], [39]. Enzyme stock concentrations and specific activities are the same as previously reported [20].

### Anti-33-mer moAbs

The A1 moAb, G12 moAb and their derived Horseradish Peroxidase (HRP)-conjugated moAb (A1-HRP and G12-HRP) were used in this study [23]. A1, G12 and A1-HRP moAb concentrations are 3.6 mg/mL, 1 mg/mL and 0.6 mg/mL, respectively. G12-HRP moAb concentration is the same as previously reported [23].

### Determination of the moAbs A1 and G12 recognition sequence within the 33-mer peptide

In order to determine the A1 and G12 recognition sequence, several fragments of DNA codifying for peptides representing the whole 33-mer sequence were constructed by synthesis of the following overlapping oligonucleotides: Leu57-Pro63, 5′-GATCTGCAGTTACAACCGTTTCCGTA-3′, and 5′-AGCTTACGGAAACGGTTGTAACTGCA-3′; Pro63-Tyr69, 5′-GATCCGCAGCCACAACTGCCGTATTA-3′, and 5′-AGCTTAATACGGCAGTTGTGGCTGCG-3′; Gln64-Pro70, 5′-GATCAGCCACAACTGCCGTATCCGTA-3′, and 5′- AGCTTACGGATACGGCAGTTGTGGCT-3′; Pro65-Gln71, 5′-GATCCACAACTGCCGTATCCGCAATA-3′, and 5′- AGCTTATTGCGGATACGGCAGTTGTG-3′; Gln66-Pro72, 5′-GATCAACTGCCGTATCCGCAACCATA-3′, and 5′-AGCTTATGGTTGCGGATACGGCAGTT-3′; Leu67-Gln73, 5′-GATCTGCCGTATCCGCAACCACAGTA-3′, and 5′-AGCTTATTATGGTTGCGGATACGGCA-3′; Pro68-Leu74, 5′-GATCCGTATCCGCAACCACAGTTGTA-3′, and 5′-AGCTTACAATTATGGTTGCGGATACG-3′; Pro82-Phe89, 5′-GATCCGTATCCTCAACCGCAACCGTTTTA-3′, and 5′-TAAAACGGTTGCGGTTGAGGATACG-3′. These synthetic DNAs were designed according to the typical codon usage of *E. coli* to maximize the expression. The resultant synthetic DNA fragments were assembled by hybridization, and cloned into the *Bam*HI-*Hin*dIII digested pALEXb plasmid (Biomedal S.L., Sevilla, Spain). This plasmid contained the Pm promoter of the Cascade expression system [40], [41] and the C-LYTAG for affinity purification of fusion peptides to a choline binding domain (Biomedal S.L., Sevilla, Spain). The resultant pALEXb-derivative plasmids were checked by sequencing, and transformed into the Cascade expression host *E. coli* REG1 (Biomedal S.L., Sevilla, Spain). A plasmid for the expression of a C-LYTAG-33mer fusion was constructed in a previous work [23] and used in this study as positive control. Cultures were then induced by addition of 2 mM salicylate and incubated 5 h at 30°C. The soluble protein extracts were used for the determination of the A1 and G12 recognition sequence by indirect ELISA.

### Preparation of gliadin solution

Gliadin (Sigma, St. Louis, Missouri, U.S.A.) was prepared in 60% (v/v) aqueous ethanol at 1 mg/mL.

### Preparation of prolamins from wheat, barley, rye, oat, corn, and rice flours

Prolamins from wheat, barley, rye, oat, corn, and rice flours were extracted by mixing 0.5 g of flour with 5 mL 60% (v/v) ethanol in a rotary shaker for 1 h at room temperature. The suspension was then centrifuged at 2,500 g for 10 min; the supernatant was separated, and protein concentration was measured by the Bradford method. The required concentration of these samples was prepared in 0.02 M PB containing BSA (5 µg/mL) before use in immunoassays.

### Indirect enzyme-linked immunosorbent assay

A Maxisorp microtiter plate (Nunc, Roskilde, Denmark) was coated overnight at 4°C with 100 µL/well of the appropriate antigen (C-LYTAG-33-mer protein, gliadin, hordein, secalin, avenin, oryzein, zein or soluble protein extracts containing peptide fusions) serially diluted with 0.1 M of Na_2_CO_3_-NaHCO_3_ (pH 9.6). After washing with PBS containing 0.05% Tween-20 (washing buffer), 300 µL of blocking buffer (washing buffer supplemented with 5% non-fat dry milk) was added to each well, and incubated for 1 h at room temperature. Plates were incubated for 1 h at room temperature with the G12 moAb (1∶1,000 in the blocking solution), A1 moAb (1∶500 in the blocking solution) or 6B5L1 moAb (1∶1,000 in the blocking solution). After washing, goat anti-mouse IgG-peroxidase antibody (Biomedal S.L., Sevilla, Spain) was added (1∶2,000 in the blocking solution). Plates were washed again, and the substrate solution (TMB liquid substrate system, Sigma, St. Louis, Missouri, U.S.A.) was added. After 30 min dark incubation, the reaction was stopped with 1 M sulfuric acid. Absorbance at 450 nm was measured in the microplate reader UVM340 (Asys Hitech GmbH, Eugendorf, Austria).

### Competitive enzyme-linked immunosorbent assay

G12 competitive ELISA was carried out according to Morón et al. [23]. For the A1 competitive ELISA, Maxisorp microtiter plates (Nunc, Roskilde, Denmark) were coated with 100 µL/well of Sigma gliadin solution (5 µg/mL) in 0.1 M of Na_2_CO_3_-NaHCO_3_ (pH 9.6), and incubated overnight at 4°C. Plates were washed with washing buffer and blocked with blocking solution for 1 h at room temperature. Gliadin and samples serially diluted in PBS containing 3% BSA (100 µL) and 100 µL solution of A1 moAb conjugated to HRP (1∶10,000 in PBS containing 3% BSA) were preincubated 3 h at room temperature with gentle mixing, and then added to the wells. After 40 min incubation at room temperature, the plates were washed, and 100 µL per well of substrate solution (TMB liquid substrate system, Sigma, St. Louis, Missouri, U.S.A.) was added. After dark incubation for 30 min at room temperature, color development was stopped with 1 M sulfuric acid (100 µL per well), and the absorbance was measured at 450 nm (microplate reader UVM340, Asys Hitech GmbH, Eugendorf, Austria).

### Western blot reactivity of the A1 moAb

For the Western blot of cereal extracts, after one-dimensional sodium dodecyl sulfate polyacrylamide gel electrophoresis (SDS-PAGE), proteins were electrotransferred on to polyvinylidene difluoride (PVDF) membranes, incubated directly with A1-HRP moAb, and developed by ECL Western Blotting Analysis System immunodetection (Amersham Biosciences, Little Chalfont, Buckinghamshire, U.K.).

### Whole-wheat bread digests and indirect competitive ELISA for 33-mer in digests

Whole-wheat bread digests were carried out as previously described [20]. Briefly, whole-wheat bread (1 g) presoaked in specified amounts of EP-B2 and SC PEP (units of activity/mg gluten protein) was added to a 37°C 0.01N HCl solution containing 0.6 mg/mL pepsin over a course of 15 min, resulting in a final pH of ∼4.5 and a final measured gluten protein concentration of ∼15 mg/mL. After this addition phase, the simultated gastric digests were incubated for 60 min at 37°C, quenched by boiling 5 min, and stored at −20°C.

The concentration of 33-mer or related epitopes remaining in each digest was determined by an indirect competitive ELISA using moAb A1. On day 1 of the procedure, synthetic 33-mer was diluted to 1.6 µg/mL in coating solution (50 mM sodium carbonate/bicarbonate buffer, pH 9.6, 0.02% NaN_3_) and 100 µL/well was incubated overnight at 4°C in 96-well microtiter plates (Nunc Maxisorp). Synthetic 33-mer standards (0.00625–51.2 µg/mL) or bread digest dilutions (1∶10–1∶163,840) were prepared in StartingBlock T20 (TBS) blocking buffer (Pierce). An equal volume of A1 moAb diluted 1∶5,000 in blocking buffer was added to each standard or sample dilution, and these mixtures were incubated overnight at 4°C. Final dilutions in mixtures were: 1∶10,000 A1 moAb and either 0.003125–25.6 µg/mL 33-mer standards or 1∶20–1∶327,680 digest dilutions. On day 2, antigen-coated plates were washed thrice with washing buffer prior to blocking and between all subsequent steps. Plates were blocked with 200 µL/well blocking buffer for 1 h at room temperature. MoAb/standard and moAb/sample preincubations were added to the wells in triplicate (100 µL/well) and incubated 3 h at room temperature. Goat anti-mouse IgG-alkaline phosphatase conjugate (Chemicon) was diluted 1∶1,000 in blocking buffer and 200 µL/well was incubated 3 h at room temperature. Freshly prepared substrate solution (5 mg/mL pNPP, 50 mM sodium carbonate/bicarbonate buffer, pH 9.8, 1mM MgCl_2_, 0.02% NaN_3_) was added (200 µL/well) and the absorbance at 405 nm was measured every 6 seconds for 5 minutes. The initial rate (mA_405_/min) of p-nitrophenolate production in each well was determined from 31 datapoints. For each digest, the initial rates were plotted against the log_10_ of the corresponding digest dilutions, yielding a sigmoidal curve; the standard curve was similarly processed. The concentration of 33-mer in each digest was determined from those digest dilutions yielding initial rates within the linear region of the 33-mer standard curve. As performed (i.e. at a minimal digest dilution of 1∶20), our assay's lower limit of quantitation 15.3 µg/mL 33-mer in the digest.

### Statistical analysis

Peptide and prolamin curves were obtained by plotting percentage of maximum absorbance against logarithm of antigen concentration. The software package Sigma Plot 9.0 (Systat Software, Inc., Point Richmond, CA, U.S.A.) was used to calculate the IC50. The limit of detection was defined here as reagent blank-3x standard deviations of reagent blank. The limit of quantification was defined here as reagent blank-10x standard deviations of reagent blank.

The CR was calculated as follows: (IC50 of the antigen for which the moAb was raised/IC50 of each antigen assayed)X100.

The reproducibility (inter-plate variability) was assessed by measuring the standard curve for different gliadin samples on two separate ELISA plates on different days. The reproducibility (intra-plate variability) was calculated by measuring the standard curve eight times for the same gliadin sample on a single ELISA plate. The coefficient of variation was calculated as follows: standard deviation/mean×100.
